# Murine Gut Microbiome Association With *APOE* Alleles

**DOI:** 10.3389/fimmu.2020.00200

**Published:** 2020-02-14

**Authors:** Ishita J. Parikh, Janice L. Estus, Diana J. Zajac, Manasi Malik, Juan Maldonado Weng, Leon M. Tai, George E. Chlipala, Mary Jo LaDu, Stefan J. Green, Steven Estus

**Affiliations:** ^1^Department of Physiology, College of Medicine, University of Kentucky, Lexington, KY, United States; ^2^Sanders-Brown Center on Aging, University of Kentucky, Lexington, KY, United States; ^3^Department of Anatomy and Cell Biology, College of Medicine, University of Illinois at Chicago, Chicago, IL, United States; ^4^Research Resources Center, University of Illinois at Chicago, Chicago, IL, United States

**Keywords:** Alzheimer's, microbiome, *APOE*, resistant starch, cladogram

## Abstract

**Background:** Since *APOE* alleles represent the most impactful genetic risk factors for Alzheimer's disease (AD), their differential mechanism(s) of action are under intense scrutiny. *APOE4* is robustly associated with increased AD risk compared to the neutral *APOE3* and protective *APOE2*. *APOE* alleles have also been associated with differential inflammation and gastrointestinal recovery after insult in human and murine studies, leading us to hypothesize that *APOE* alleles impact the gut microbiome.

**Methods:** To assess this hypothesis, we compared 16S ribosomal RNA gene amplicon-based microbiome profiles in a cohort of mice that were homozygous for *APOE2, APOE3, or APOE4*, and included both males and females as well as carriers and non-carriers of five familial AD (5xFAD) mutations. Fecal samples were analyzed from mice at 4 and 6 months of age. *APOE* genotype, as well as sex and 5xFAD status, was then tested for influence on alpha diversity (Shannon H index) and beta diversity (principal coordinate analyses and PERMANOVA). A Random Forest analysis was used to identify features that predicted *APOE*, sex and 5xFAD status.

**Results:** The richness and evenness (alpha diversity) of the fecal microbiome was not robustly associated with *APOE* genotype, 5xFAD status or sex. In contrast, microbial community composition (beta-diversity) was consistently and strongly associated with *APOE* genotype. The association between beta-diversity and sex or 5xFAD status was less consistent and more modest. Comparison of the differences underlying *APOE* effects showed that the relative abundance of multiple bacterial taxa was significantly different as a function of APOE genotype.

**Conclusions:** The structure of the gut microbiome was strongly and significantly associated with *APOE* alleles in this murine model. Further evaluation of these findings in humans, as well as studies evaluating the impact of the APOE-associated microbiota on AD-relevant phenotypes in murine models, will be necessary to determine if alterations in the gut microbiome represent a novel mechanism whereby *APOE* genotype impacts AD.

## Introduction

Apolipoprotein E (*APOE*) alleles constitute a major genetic risk factor for Alzheimer's disease (AD); relative to the common *APOE3* allele, *APOE4* strongly increases AD risk while *APOE2* reduces AD risk [reviewed in (1, 2)]. The primary mechanism(s) whereby *APOE* genetics influence AD risk are not resolved although apoE alleles have been implicated in differential amyloid-beta (Aß) clearance, Aß aggregation, astrocyte stress and brain cholesterol homeostasis (3–8). Elucidating differential actions of apoE alleles could provide insights into AD.

Several reports have suggested a relationship between apoE, the gut microbiome and intestinal health. First, *APOE*-deficient mice display microbiome differences relative to wild-type mice (9). Second, *APOE*-targeted replacement (TR) mice have genotype-dependent differences in response to gastrointestinal insult, i.e., *APOE4* mice were more resistant to *Cryptosporidium* infection than *APOE3* mice (10). Third, *APOE* allelic effects on gut health are not limited to mice but have also been observed in humans; *APOE4* was associated with better defense against childhood diarrheal diseases in a third world environment, resulting in enhanced nutritional and cognitive outcomes (11–13). Fourth, a recent study suggested the presence of microbiome differences in a comparison of *APOE3* and *APOE4* TR mice (14). The mechanism(s) whereby apoE alleles may influence the gut microbiome are unclear, although *APOE4* has been associated with a greater inflammatory response to a microbiome product, lipopolysaccharide (LPS), in both humans and mice (15, 16).

Several reports have found that altering the gut microbiome impacts Aß-related pathology in murine models, i.e., Aß burden is reduced in Aß protein precursor mice maintained in a gnotobiotic environment, treated with broad-spectrum antibiotics, or fed bacterial “cocktails” (17–20). Here, we confirm and extend to this emerging scientific area by reporting that murine gut microbiome profiles are associated with *APOE* genetics in a comparison of homozygous *APOE2, APOE3*, and *APOE4* mice.

## Materials and Methods

### Mice

As described previously, EFAD mice are homozygous for *APOE2, APOE3*, or *APOE4* and heterozygous for 5xFAD (3, 21–26). Briefly, these mice were derived by crossing the *APOE*-TR mice to the commonly used 5xFAD mice. EFAD mice develop plaques in the subiculum and cortex, with E4FAD mice having significantly more plaques than the E3FAD and E2FAD mice at 4 and 6 months of age (21). In the subiculum, microgliosis was comparable between mice with different APOE isoforms (3). This study used fecal samples of convenience from on-going studies that included both carriers and non-carriers of the 5xFAD mutations. Mice were housed separately by sex, with 2–5 mice per cage [average of 3.1 ± 1.0 (mean ± SD)]. Feces were obtained from 139 mice at 4 months of age and 91 mice at 6 months of age (Table 1). A subset of the mice contributed feces at both time points including 11 *APOE2*, 11 *APOE3*, and 18 *APOE4* animals. Feces were obtained by placing each mouse into a Styrofoam cup with a new cup used for each mouse. Upon defecation, the fecal pellet was immediately flash frozen on dry ice followed by storage at −80°C until DNA isolation.

**Table 1:** Number of animals by *APOE* genotype, 5xFAD status, sex and age that generated fecal microbiome samples for this study [Male (M), Female (F)].

***APOE***	**F – 5xFAD**	**M – 5xFAD**	**F + 5xFAD**	**M + 5xFAD**	**Total – 5xFAD**
**4 MONTHS**					
2	14	9	6	4	33
3	11	15	20	9	55
4	16	15	12	8	51
**6 MONTHS**					
2	10	7	5	2	24
3	4	6	2	4	16
4	8	20	12	11	51

### Microbiome Analysis

Fecal DNA was isolated by using a PowerSoil DNA extraction kit (Mo Bio Laboratories). The V4 variable region of microbial 16S ribosomal RNA (rRNA) genes was PCR-amplified by using target-specific primers that contained bar codes and linker sequences (27). PCR reaction conditions included an initial denaturation step of 30 s at 98°C, followed by 28 cycles of 10 s at 98°C, 15 s at 60°C, 30 s at 72°C, and a final elongation step of 7 min at 72°C. The PCR master mix (20 μl volume) contained 100 ng of DNA template, 0.5 μM forward and reverse primers, Phusion Hot Start DNA polymerase and high-fidelity buffer (New England Biolabs), dNTPs and distilled water. Samples were pooled in equimolar ratio for sequencing (Illumina MiSeq, University of Kentucky Advanced Genetic Technologies Center). T Forward and reverse reads were merged by using the software package PEAR (28). Merged reads were trimmed to remove ambiguous nucleotides, primer sequences, and trimmed based on quality threshold of *p* = 0.01. Reads that lacked either primer sequence or were shorter than 225 bp were discarded. Chimeric sequences were identified and removed using the USEARCH algorithm with a comparison to the SILVA v132 reference database (29, 30). Amplicon sequence variants (ASVs) were identified using DADA2 (31). The representative sequences for each ASVs were then annotated using the Naïve Bayesian classifier included in DADA2 with the SILVA v132 training set. A multiple sequence alignment of the representative sequences was generated using PyNAST with GreenGenes 80% OTUs as a template alignment (32, 33). The multiple sequence alignment was then used to generate a phylogenetic tree using FastTree (34).

This sequencing effort yielded 3.05 million reads. Eight samples with fewer than 3,000 reads each were discarded. The read counts across *APOE* genotypes were similar, i.e., the average read count for *APOE2* samples was 14,601 ± 555 (mean ± SD), *APOE3* samples was 14,622 ± 517 and *APOE4* was 13,798 ± 485. In our primary analysis using the software package MicrobiomeAnalyst (35), samples were rarified to the minimum library size (5,364 for the 4-month sample set, and 4,020 for the 6-month sample set), low abundance amplicon sequence variants (ASV)s were removed, i.e., only ASVs with ≥ 4 counts in ≥ 10% of the samples were retained, and low variance ASVs were also removed, i.e., those with an inter-quantile range <10% (35). These corrections reduced the number of ASVs from 268 to 77 for the 4-month samples and 76 for the 6-month samples. A centered log-ratio transformation was used for normalization.

Alpha-diversity was assessed by using the Shannon H diversity index (36) with statistical significance determined by Mann–Whitney (sex and 5xFAD status) or Kruskal–Wallis (*APOE* genotype) non-parametric tests. Beta-diversity was assessed by using Principal Coordinates Analysis (PCoA) of Bray-Curtis matrices with statistical significance determined by Permutational Multivariate Analysis of Variance (PERMANOVA) (37). In a secondary evaluation, beta diversity was evaluated by using unweighted and weighted UniFrac analyses (see Supplemental Methods and Figures).

Bacteria associated with *APOE* were identified by a linear discriminant analysis effect size (LefSe) approach and plotted as a cladogram (38). This comparison used a one-against-all approach with cut-off values of 0.001 for the Kruskal–Wallis alpha and 2.0 for the linear discriminant analysis. Additional insights regarding bacteria associated with *APOE*, sex or 5xFAD transgene status were gained by using a Kruskal–Wallis test (*APOE*) or Mann–Whitney test (sex or 5xFAD carrier status, with a false discovery rate (FDR) approach used to correct for multiple testing (35). To identify features that were predictive of meta variables, we used a Random Forest analysis which included all ASVs (77 for 4-month dataset, 76 for 6-month dataset) with stipulations of 5,000 trees and the number of features used at each split of the decision tree (mtry parameter) set at nine, i.e., the square root of the number of features post filtering (39, 40). Raw sequence data files were submitted in the Sequence Read Archive (SRA) of the National Center for Biotechnology Information (NCBI). The BioProject identifier is PRJNA556445.

## Results

**Figure 1:**
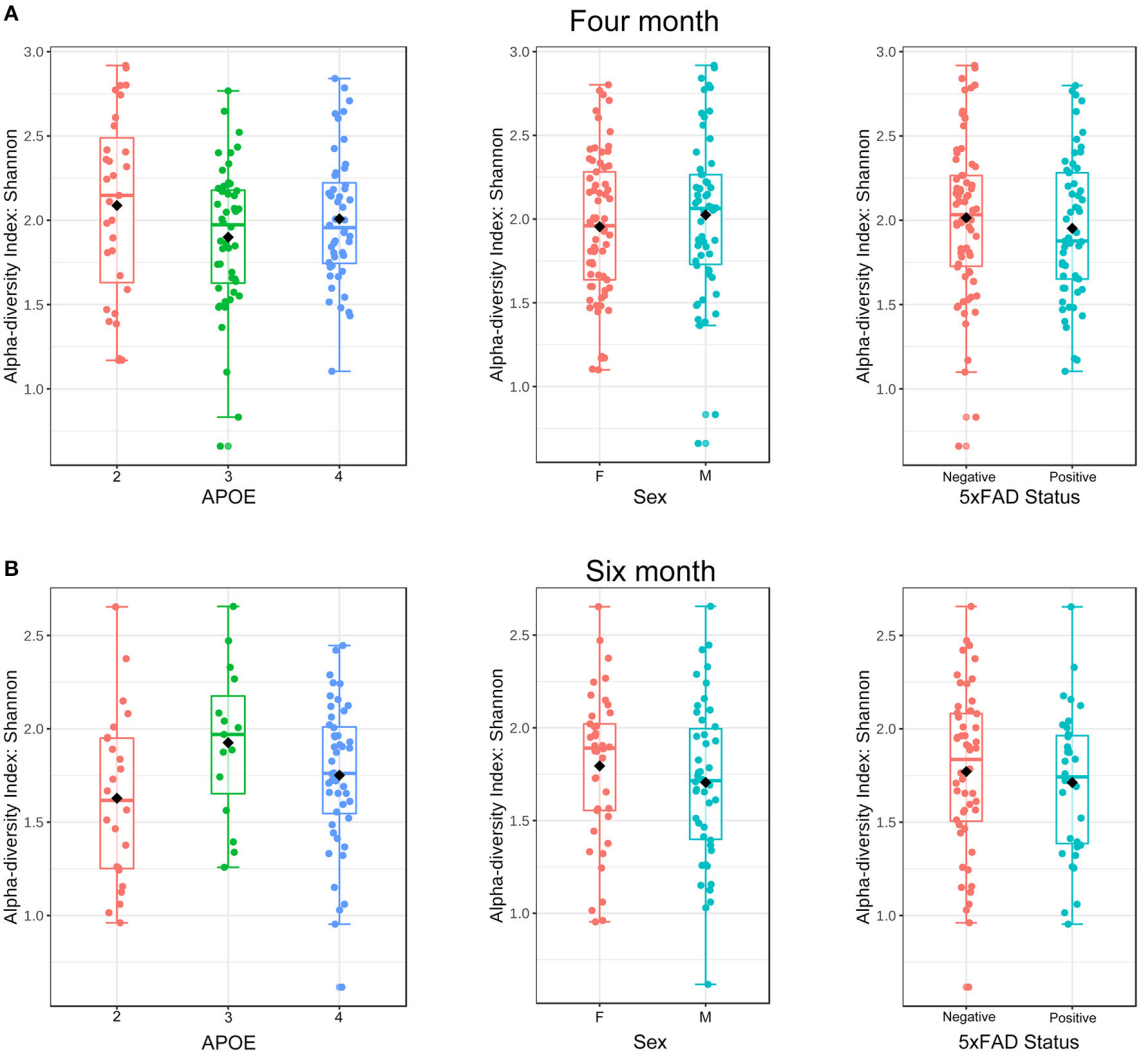
Alpha-diversity at the ASV level as a function of *APOE*, sex and 5XFAD status at 4 **(A)** and 6 **(B)** months of age as assessed using the Shannon H index. These samples from 4 to 6 months were rarified to 5,364 and 4,020 ASVs, respectively.

To investigate the hypothesis that *APOE* alleles are associated with the gut microbial community structure, we began by comparing alpha (within sample)—diversity as assessed by the Shannon H index, a measure of taxon richness and evenness. We also evaluated sex and 5xFAD status. No association between alpha-diversity and *APOE*, sex or 5xFAD status was detected at either 4 or 6 months (Figure 1, Table 2).

**Table 2:** Microbiome alpha-diversity (Shannon H index) was not associated with *APOE*, sex or 5xFAD status.

**Variable**	***P*-value (4 months)**	***P*-value (6 months)**
*APOE*	0.285	0.100
Sex	0.390	0.292
5xFAD status	0.358	0.428

*P-values were determined using non-parametric Mann-Whitney tests for sex and 5xFAD status and Kruskal–Wallis tests for APOE*.

**Figure 2:**
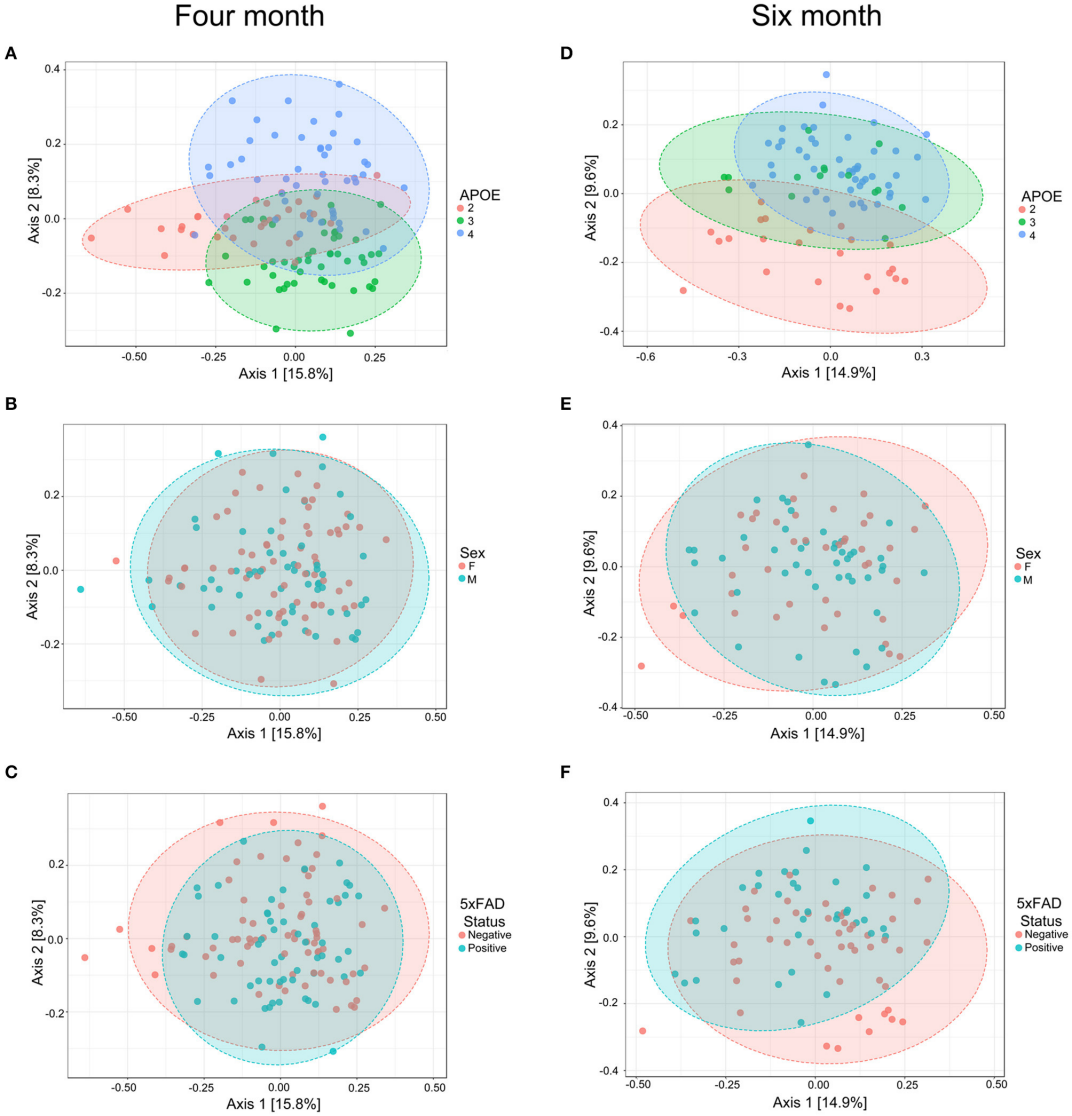
PCoA of fecal microbiome profiles in 4-month-old **(A–C)** and 6-month-old **(D–F)** mice shows a separation as a function of *APOE* relative to sex or 5xFAD status. Ellipses represent 95% confidence.

We next evaluated whether *APOE* genetics were a significant source of beta (between sample) diversity, a measure of microbial communities based on their composition. Results were visualized by using PCoA based on Bray-Curtis distance matrices (41–44), a robust effect was observed for *APOE* relative to sex or 5xFAD status (Figure 2). When these findings were analyzed by PERMANOVA as well as unweighted and weighted UniFrac analyses, we found that microbiome profiles were consistently and robustly associated with *APOE* genotype at both 4 and 6 months (Figure 2, Table 3, Figure S1, Tables S1.1–S1.6). Beta diversity was not consistently associated with sex or 5xFAD status (Table 3, Tables S1.3–S1.6). Since *R*^2^ denotes the percentage of the dissimilarity that is explained by a term, comparison of the *R*^2^ values shows that *APOE* has a larger impact than sex or 5xFAD status on the overall composition of the microbial community (Table 3).

**Table 3:** Beta diversity shows a consistent and robust association with *APOE* genotype as assessed with three different analyses.

**Variable**	**Bray–Curtis PERMANOVA**			**Unweighted unifrac**			**Weighted unifrac**		
	***P*-value**	***R*^**2**^**	***F*-value**	***P*-value**	***R*^**2**^**	***F*-value**	***P*-value**	***R*^**2**^**	***F*-value**
**4 MONTH DATASET**									
*APOE*	<0.001	0.089	6.063	<0.001	0.222	17.908	<0.001	0.200	15.778
Sex	0.350	0.007	0.931	0.002	0.025	4.028	0.003	0.029	4.634
5xFAD	0.398	0.007	0.888	0.195	0.008	1.368	0.091	0.011	1.791
**6 MONTH DATASET**									
*APOE*	<0.001	0.089	6.063	<0.001	0.256	14.529	<0.001	0.196	11.789
Sex	0.350	0.007	0.931	0.008	0.025	2.820	<0.001	0.061	7.374
5xFAD	0.398	0.007	0.888	0.024	0.020	2.212	0.120	0.015	1.786

*Significant effects of sex or 5xFAD status were not consistently observed. The PERMANOVA results were derived from 999 permutations*.

**Figure 3:**
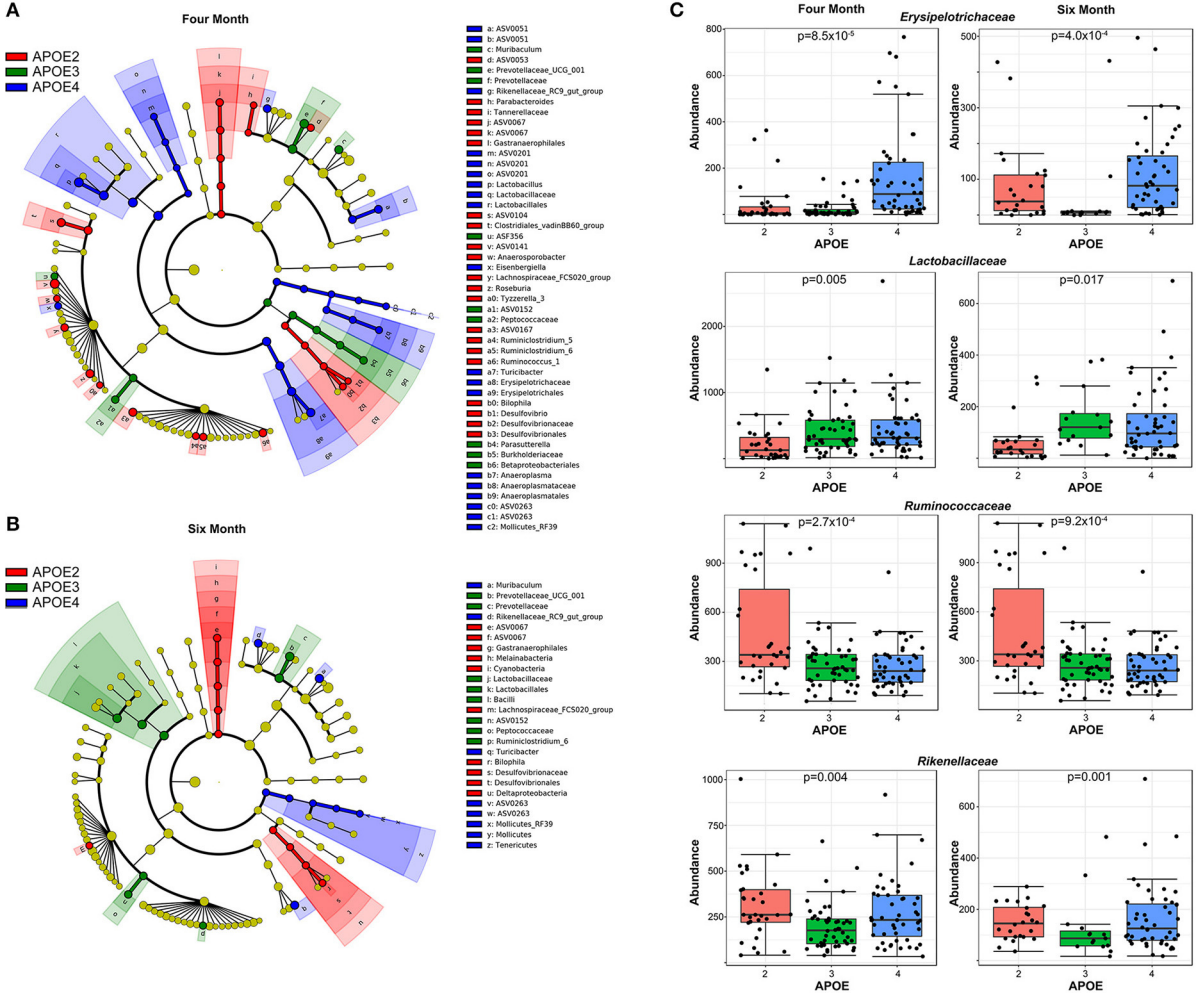
*APOE* genotype is associated with multiple bacteria. A LefSe analysis shows that the effects of *APOE* genotype are fairly broad across the microbiome **(A)**. Quantification of representative bacterial families with significant differences with *APOE* in mice at both 4 and 6 months of age are shown **(B,C)**. The *p*-values have been corrected by using an FDR approach.

To visualize the phylogenetic relatedness of bacteria significantly associated with *APOE*, we performed a LefSe analysis. The effects of *APOE* genetics appeared broad, as multiple bacterial classes showed differences with *APOE* genetics (Figure 3A). Bacteria associated with *APOE* at both 4 and 6 months included *Prevotellaceae, Rikenellaceae, Gastranaerophilales, Lactobacillaceae, Peptococcaceae, Turicibacter* of the *Erysipelotrichaceae* family, Desulfovibrionales, and Mollicutes of the Tenericutes phylum (Figure 3A). We performed further classical univariate analysis using a Kruskal-Wallis test for *APOE and* a Mann-Whitney test for sex and 5xFAD status with an FDR correction set to *p* < 0.05. In the 4-month-old animals, the analyzed dataset had 22 bacterial families, of which 15 had a significantly different relative abundance by *APOE* genotype (Table S2.1). Similarly, at 6 months, the relative abundance of seven of the 22 families was significantly different by *APOE* genotype (Table S2.1). Among the significant families, six were significant at both 4 and 6 months (Table S2.1, representative examples shown in Figures 3B,C). Relative to these family associations with *APOE*, few bacterial families were associated with sex or 5xFAD status. At 4 months, no families were significantly associated with sex or 5xFAD status, while at 6 months, only one family was associated with sex (*Prevotellaceae*) and no families were associated with 5xFAD status (Tables S3.1–S4.2).

**Figure 4:**
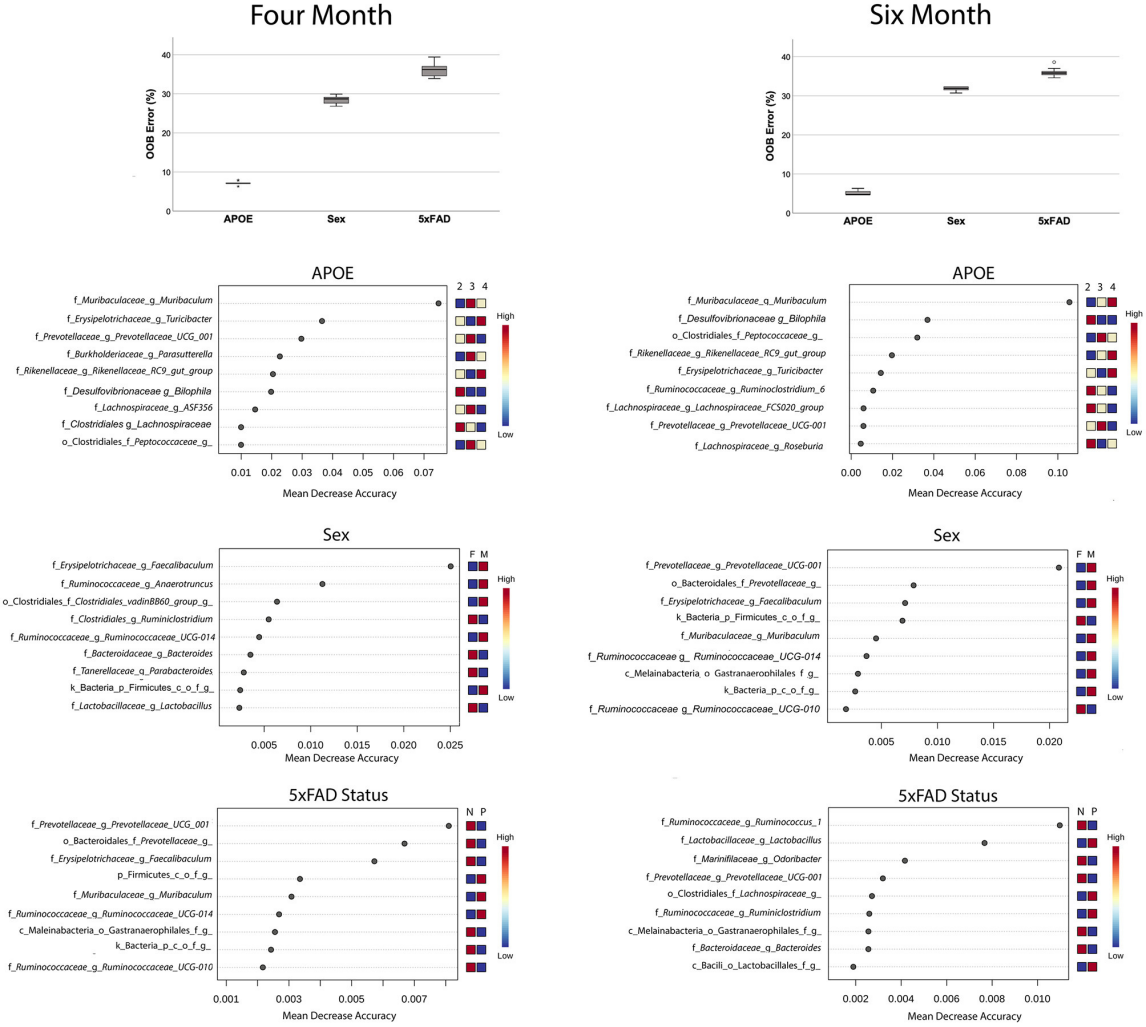
Random Forest analyses find that microbiome features accurately predict *APOE* genotype relative to sex or 5xFAD status (box plots). Features that predict *APOE* genotype, sex and 5xFAD status are shown the features plots below. Note the differences in the abscissa scale for the graphs of *APOE* vs. sex and 5xFAD status, which reflects the greater prediction accuracy of the bacteria for APOE genotype.

Lastly, we evaluated the extent that individual families were predictive of *APOE* genotype, sex or 5xFAD status by using a Random Forest analysis. The accuracy of Random Forest classifiers is defined by the out of the bag (OOB) error, which correlates with the frequency of incorrect predictions. This approach found that microbiome profiles were highly efficient at classifying by *APOE* and relatively inefficient when predicting sex or 5xFAD status (Figure 4). Although this analysis identified the same bacterial families that were identified as associated with *APOE* genetics by the LefSe approach (Figure 3), this Random Forest analysis provided the relative contributions to the bacteria to the accuracy of the prediction. The most robust predictor was Muribaculaceae (previously known as S24–7), which is abundant in the murine but not human gut microbiome and, indeed, has been shown to out-compete transplanted human gut microbiota in the murine gut (45, 46).

## Discussion

The primary finding reported here is a gut microbiome association with *APOE* genetics in the widely used *APOE*-TR murine model. More specifically, an analysis of microbial community structure, i.e., beta-diversity, showed a robust association of the microbiome with *APOE* alleles. As such, these findings confirm and extend a recent report that beta-diversity was associated with *APOE* genetics in a comparison that involved only *APOE3* and *APOE4* mice (14). Overall, these findings contribute to our understanding of multi-system differential *APOE* allelic effects in areas impacted by microbiome, which could be broad and includes gut health, inflammation, lipid metabolism and, possibly, AD.

The identities of *APOE*-associated microbial families may provide insights into whether the microbiome contributes to *APOE*-associated actions. Since the functions associated with bacterial families are still emerging, this discussion focuses on bacterial families with greater characterization. *APOE4* was associated with improved outcomes relative to *APOE3* after gastrointestinal insults such as *Cryptosporidium* infection and malnutrition in mice and non-specified diarrheal disease in humans (10–13, 47, 48). Here, we found that the relative abundance of bacteria from the *Lactobacillaceae* family was higher in *APOE4* and lower in *APOE2* mice at both 4 and 6 months of age (Figures 3B,C). This increase in *Lactobacillus* species may contribute to *APOE4* beneficial effects in the gut because *Lactobacillus* has been associated with improved gut health with regards to *Cryptosporidium* or fungal infections specifically (49, 50) and gut health in general [reviewed in (51, 52)]. The protective actions of *Lactobacillus* family members have been ascribed to lactic acid secretion, resulting in localized acidification, as well as hydrogen peroxide secretion, resulting in a microenvironment that antagonizes colonization by other species (53). Hence, the improved gastrointestinal health observed with *APOE4* relative to *APOE2* in mice and humans may reflect, in part, an increase in the relative abundance of *Lactobacillaceae*. Another bacterial family that was increased with *APOE4* was *Erysipelotrichaceae* which has been shown to be induced by a high fat diet (54, 55). Whether this family may contribute to the *APOE4* association with fatty acid metabolism and better response to a high fat meal (56, 57) will be examined in future studies.

Bacterial taxa that showed a relative abundance pattern that was highest in *APOE2* included members of the *Ruminococcaceae* and *Rikenellaceae* families (Figure 3, Tables S2.1–S2.2). Relevant to these findings, we note that others have reported that the relative abundance of members of the *Ruminococcaceae* family may be increased in humans with the *APOE2* allele (14). Interestingly, the *Ruminococcaceae and Rikenellaceae* families have been reported to increase in relative abundance in humans and mice fed a diet high in resistant starch (58–61). The induction of these bacteria by resistant starch reflects that resistant starches [classes RS1, RS2, and RS3 (RS4 is non-digestible)] are not digested in the small intestine but rather pass through into the large intestine. Within the large intestine, resistant starch digestion by bacteria like *Ruminococcaceae* influences bacterial proliferation and thereby alters microbiome community profiles. Indeed, members of the *Ruminococcaceae* family in particular are termed “keystone” for the degradation of resistant starch (60). Further digestion of resistant starch metabolites in the large intestine generates short chain fatty acids (SCFA)s, which affect human health in general [reviewed in (62, 63)] and have been reported to promote microglial maturation and function in particular (64). Considering these findings relative to AD, we propose a tentative model wherein (i) *APOE2* is associated with an increase in the relative abundance of microbiome bacteria like *Ruminococcaceae*, relative to *APOE3* and *APOE4*, (ii) this shift in bacterial profile increases the efficiency of resistant starch metabolism to SCFAs and (iii) this increase in SCFAs promotes microglial function (64) to reduce AD risk, as suggested by robust genetic evidence (65–72), [reviewed in (73, 74)]. While speculative, this model serves as a framework for future studies.

The findings reported here are generally consistent with prior related studies. Regarding *APOE* genetics and the microbiome, Tran et al. recently compared microbiota profiles in *APOE3* vs. *APOE4* mice at 4 and 18 months of age (14). Some of the findings in the 4-month old mice in this study and that of Tran et al. are consistent. For example, both studies report robust differences in beta diversity with *APOE* genetics. With regards to specific bacteria, a subset of the findings are consistent between the studies, e.g., both studies found that *Prevotellaceae* was low in *APOE4* mice and *Rikenellaceae* was low in *APOE3* mice. Other results are inconsistent, e.g., this study found that *Erysipelotrichaceae* increased with *APOE4* while Tran et al. found a decrease with *APOE4* (14). Several reports have also appeared comparing the microbiome of AD and non-AD murine models [(75–78), reviewed in (79)]. These findings have not been generally consistent, which may reflect differences in models, age of the mice, housing, vendor and/or diet (79). Here, we did not identify bacterial families that were significantly altered with 5xFAD status. Several bacteria were weakly predictive of 5xFAD status in the Random Forest analysis, including members of the *Prevotellaceae* family and a member of the *Ruminococcaceae* family (Figure 4). Consistent with a lack of statistical significance in our study, members of these families have not been associated with murine AD models in prior studies (79).

The mechanism(s) whereby *APOE* alleles may differentially modulate the gut microbiome is not known. In overview, we theorize that apoE alleles differentially modulate the basal inflammation state of intestinal macrophages, which are key regulators of innate lymphoid cells in the gut and, in turn, impact the gut microbiome. The rationale for this includes (i) *APOE4* is associated with a greater pro-inflammatory response in multiple studies, e.g., treatment with LPS results in a greater plasma cytokine response in *APOE4* positive humans, compared to *APOE4* negative individuals (16). Similarly, treatment with bacterial lipoproteins or with LPS has been reported to produce an increased cytokine response in the *APOE* murine model used here (15, 80). Within the gut, pro-inflammatory signaling from intestinal macrophages to innate lymphoid cells modulates their interactions with multiple cell types to impact the gut microbiome [(81–83), reviewed in (84, 85)]. Hence, the greater propensity of *APOE4* to promote inflammation, relative to *APOE3*, may underlie these microbiome differences.

Potential limitations of this study are several. First, since fecal samples for this study were samples of convenience obtained from mice that were used in unrelated studies on apoE actions *in vitro* and *in vivo*, these mice were not maintained in an ideal fashion for a microbiome study, e.g., used mouse bedding was not mixed among the cages to minimize environmental effects on the microbiome (86). To evaluate this possibility, we evaluated whether cage was a significant variable and found that the *APOE* genotype had a much stronger influence than cage on beta diversity (Figure S2). Second, all mice were homozygous for their *APOE* allele. Future studies should examine mice with heterozygous genotypes, as this condition is much more common in humans. Third, type-3 hyperlipoproteinemia occurs in only 10% of human *APOE2/2* individuals but is present in 100% of the *APOE2* mice (87–89). Whether this hyperlipoproteinemia may contribute to the microbiome phenotype reported here is unclear.

## Conclusions

In summary, we report a significant association between host *APOE* genotype and gut microbiome profiles in 4- and 6-month male and female mice with and without 5xFAD mutations. The taxa most strongly affected by *APOE* genotype, including bacteria from the family *Lactobacillaceae*, may contribute to differences in gastrointestinal health observed with *APOE*. The increase in *Ruminococcaceae* and related bacteria with *APOE2* may reflect an increase in resistant starch metabolism with *APOE2*, which would impact SCFA levels. Future studies that evaluate a possible interaction between sex and *APOE4* and assess the impact of the *APOE*-associated microbiome on AD-related phenotypes will be critical to assess the potential impact of the microbiome in *APOE* genetic actions.

## Data Availability Statement

The datasets generated for this study can be found in the raw sequence data files were submitted in the Sequence Read Archive (SRA) of the National Center for Biotechnology Information (NCBI). The BioProject identifier is PRJNA556445.

## Ethics Statement

The animal study was reviewed and approved by the Institutional Animal Care and Use Committee at University of Illinois-Chicago.

## Author Contributions

IP and MM purified DNA and performed PCR. JE, GC, and SG performed statistical analyses. IP, DZ, JM, LT, ML, SG, and SE analyzed data, generated, and edited the manuscript.

### Conflict of Interest

The authors declare that the research was conducted in the absence of any commercial or financial relationships that could be construed as a potential conflict of interest.
