# Supplementary material for: Murine Gut Microbiome Association With APOE Alleles
Source: Front Immunol. 2020 Feb 14;11:200. doi: 10.3389/fimmu.2020.00200 (PMC7034241; doi:10.3389/fimmu.2020.00200)
Supplement: Supplementary file 1 [file Data_Sheet_1.docx]

**Supplemental Methods, Figure S1 and Tables S1.1-S1.6**

## Weighted Unifrac Beta Diversity Analyses

Weighted UniFrac indices were calculated from the rarified ASV by using R within the phyloseq library [1]. The resulting dissimilarity indices were modelled and tested for significance with the sample covariates, i.e. *APOE* genotype, gender, and 5xFAD status, using the ADONIS test included in the vegan package [2]. Plots were generated in R using the ggplot2 library and the PCOA function in the APE library [3; 4]. This analysis was performed by the UIC Research Informatics Core.


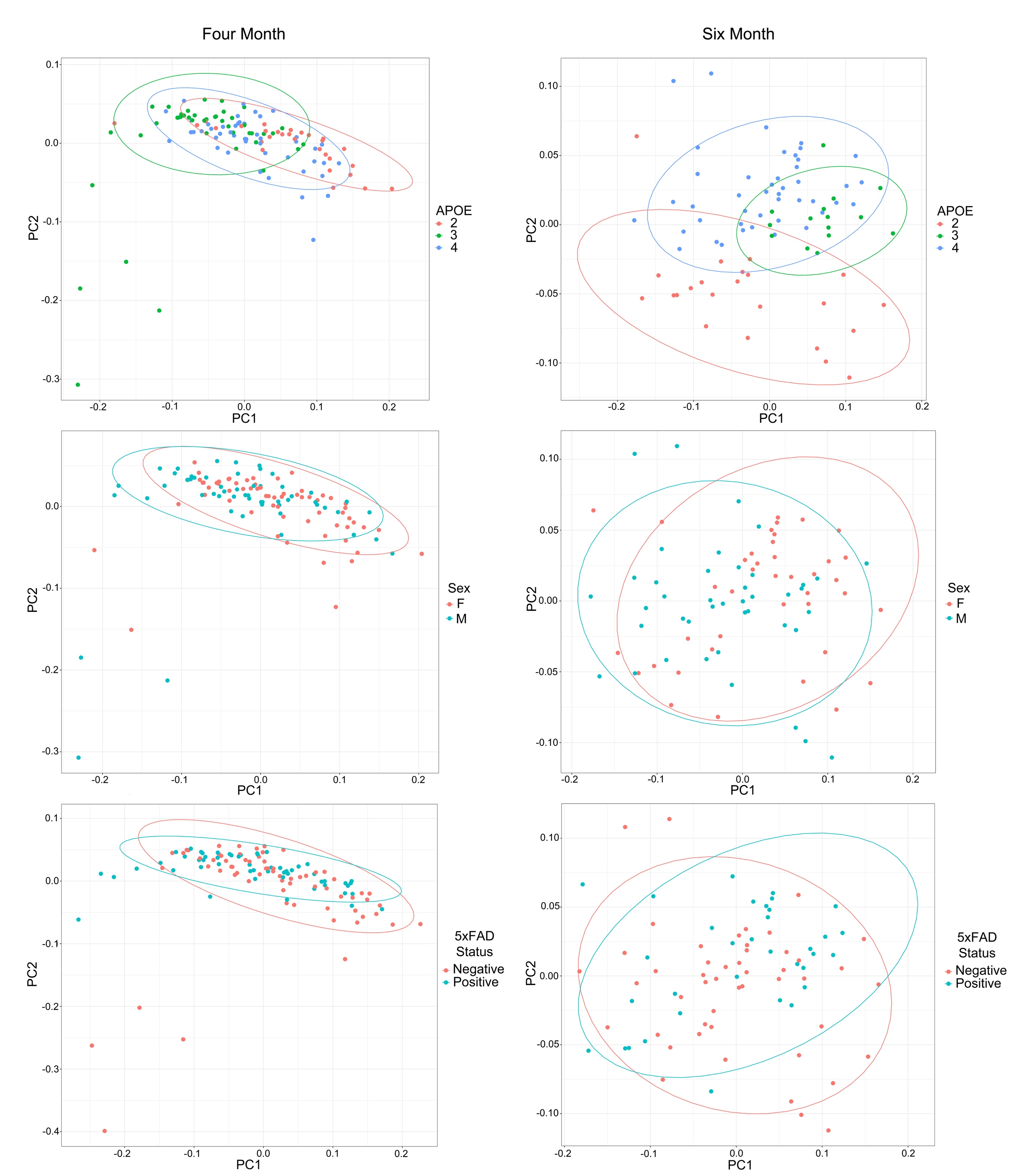


Figure S1. PCoAs of fecal microbiome beta diversity as determined by weighted UniFrac. Ellipses represent 95% confidence.

ADONIS analyses of the weighted UniFrac results support the finding that *APOE* is significantly associated with gut microbiome beta diversity. A significant association of sex with beta diversity was also observed. Possible interactions between *APOE* and either sex or 5xFAD status were not consistently significant. These findings will be probed in future studies.

==============================================================

Table S1.1 APOE and Beta Diversity at Four Months

Model: dist ~ APOE

Factor Df F.Model R^2^ Pr(>F) Signif

*APOE* 2 23.806 0.192 0.001 ***

Table S1.2 *APOE* and Beta Diversity at Six Months

Model: dist ~ APOE

Factor Df F.Model R^2^ Pr(>F) Signif

*APOE* 2 9.954 0.201 0.001 ***

Tables S1.1 and S1.2 show that the microbiome beta diversity is significantly associated with *APOE* at both four and six months.

==============================================================

==============================================================

Table S1.3 *APOE*, Sex and Beta Diversity at Four Months

Model: dist ~ APOE * Sex

Factor Df F.Model R^2^ Pr(>F) Signif

*APOE* 2 15.848 0.206 0.001 ***

Sex 1 4.246 0.0276 0.006 **

*APOE*:Sex 2 1.516 0.0197 0.125

Table S1.4 *APOE*, Sex and Beta Diversity at Six Months

Model: dist ~ APOE * Sex

Factor Df F.Model R^2^ Pr(>F) Signif

*APOE* 2 10.650 0.196 0.001 ***

Sex 1 6.5556 0.0603 0.003 **

*APOE*:Sex 2 2.4718 0.0454 0.027 *

Tables S1.3 and S1.4 show that microbiome beta diversity is significantly associated with *APOE* and sex at both four and six months.  In both cases, the effect associated with *APOE* (R^2^=0.206, 4 months and R^2^=0.169, 6 months) was considerably larger than the effect associated with sex (R^2^=0.0276, 4 months and R^2^=0.0603, 6 months).

==============================================================

==============================================================

Table S1.5 *APOE*, 5xFAD Status and Beta Diversity at Four Months

Model: dist ~ *APOE* * 5xFAD Status

Factor Df F.Model R^2^ Pr(>F) Signif

*APOE* 2 14.835 0.199 0.001 ***

5xFAD 1 1.570 0.0105 0.144

*APOE*:5xFAD 2 1.512 0.0203 0.149

Table S1.6 *APOE*, 5xFAD Status and Beta Diversity at Six Months

Model: dist ~ *APOE* * 5xFAD

Factor Df F.Model R^2^ Pr(>F) Signif

*APOE* 2 10.965 0.196 0.001 ***

5xFAD 1 1.659 0.015 0.149

*APOE*:5xFAD 2 6.277 0.112 0.001 ***

Tables S1.5 and S1.6 support the hypothesis that *APOE* is associated with beta diversity. The interaction term for *APOE* and 5xFAD status is significant at six but not four months.

==============================================================

==============================================================

## Comparison of the Impact of APOE Genotype versus Cage on Beta Diversity

To assess whether home cage is a more robust contributor to beta diversity than APOE genotype, we compared beta diversity as a function of cage number versus *APOE* status.  For this analysis, cage was included as a covariate for every cage with three or more animals. We compared *APOE2* vs *APOE4* mice to have the greatest clarity.  Analysis of beta diversity by Brey-Curtis showed that microbiota profiles were well-separated by *APOE* genotype (Figure S2).  The letters A-O label each datapoint and refer to different cages. Overall, *APOE* genetics separate microbiota by beta diversity very well while cage labels show more variation. For example, animals from cage J are at both ends of the *APOE2* demarcated microbiome but yet are well-distinct from the *APOE4* dataset. Overall, since *APOE* genotype is robust relative to cage, home cage does not appear to be a confound in this study.


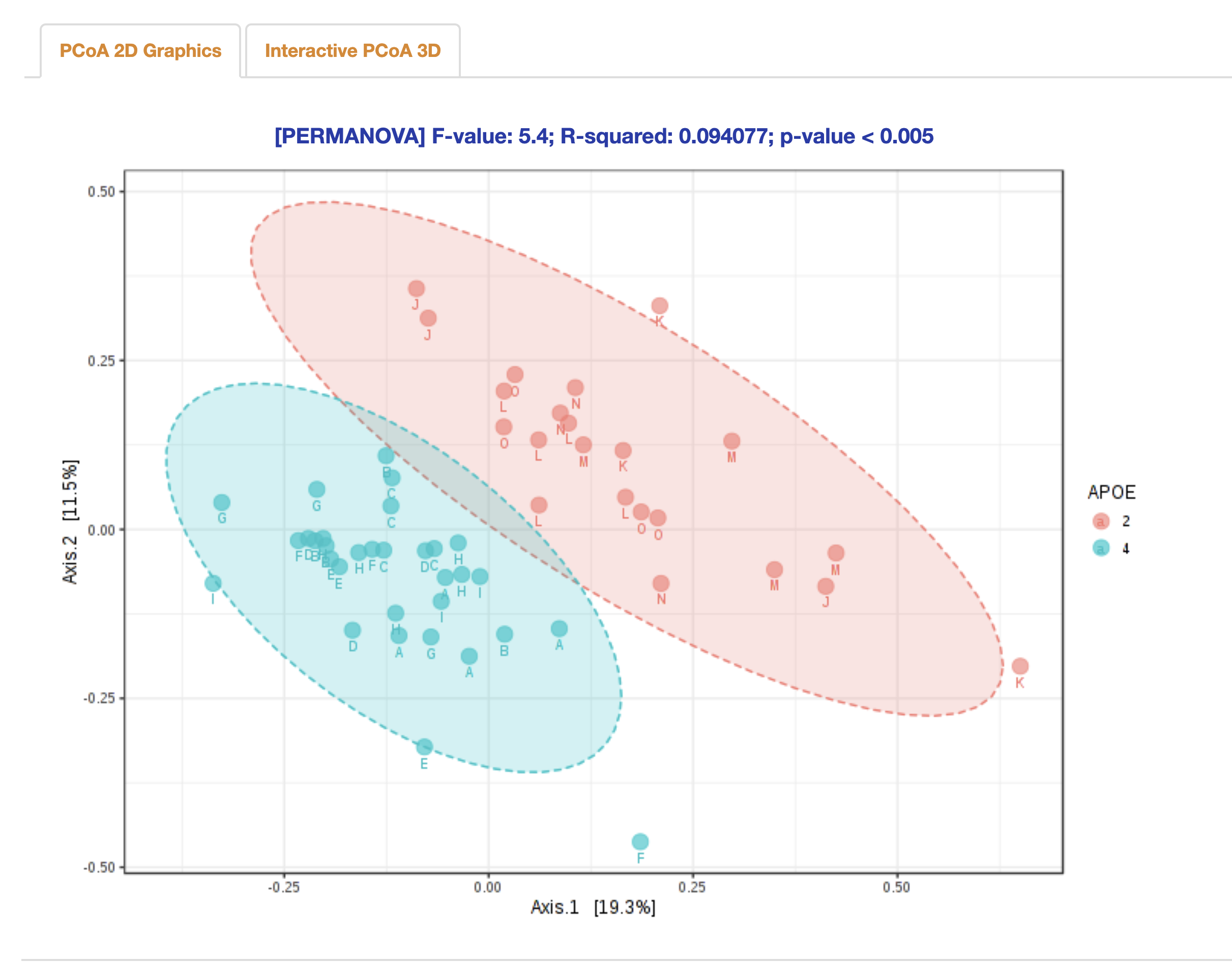


Figure S2. Home cage is not associated with microbiome beta diversity relative to APOE genotype.

References

[1] P.J. McMurdie, and S. Holmes, phyloseq: an R package for reproducible interactive analysis and graphics of microbiome census data. PloS one 8 (2013) e61217.

[2] J. Oksanen, F.G. Blanchet, R. Kindt, P. Legendre, R.B. O'Hara, G.L. Simpson, S. P., M.H.H. Stevens, and H. Wagner, Vegan: community ecology package. R package version 2.4.0 (Computer Program), 2018.

[3] E. Paradis, J. Claude, and K. Strimmer, APE: Analyses of Phylogenetics and Evolution in R language. Bioinformatics 20 (2004) 289-90.

[4] H. Wickham, ggplot2 : elegant graphics for data analysis, Springer, New York ; London, 2009.
